# *Aggregatibacter actinomycetemcomitans* and *Aggregatibacter aphrophilus* in a Kenyan Maasai Adolescent Population and Inhibition of Leukotoxic Activity by Herbal Plants Used as Part of Oral Hygiene Procedures

**DOI:** 10.3390/jcm10225402

**Published:** 2021-11-19

**Authors:** Mark Lindholm, Rolf Claesson, Arthur Kemoli, Tonnie Mulli, Jan Oscarsson, Dorte Haubek, Anders Johansson

**Affiliations:** 1 Department of Odontology, Umeå University, 901 87 Umeå, Sweden; mark.lindholm@umu.se (M.L.); rolf.claesson@umu.se (R.C.); jan.oscarsson@umu.se (J.O.); 2 Department of Paediatric Dentistry & Orthodontics, University of Nairobi, Nairobi 00100, Kenya; musakulu@gmail.com; 3 Department of Periodontology, University of Nairobi, Nairobi 00100, Kenya; mullitonnie@yahoo.com; 4 Department of Dentistry and Oral Health, Health, Aarhus University, DK-8000 Aarhus, Denmark; dorte.haubek@dent.au.dk

**Keywords:** *Aggregatibacter actinomycetemcomitans*, *Aggregatibacter aphrophilus*, leukotoxicity, herbal plants, Maasai adolescents

## Abstract

Background: A virulent genotype (JP2) of the periodonto-pathogen, *Aggregatibacter actinomycetemcomitans* (*Aa*), is widespread in North and West Africa, while its presence in East Africa has not been thoroughly investigated. This JP2 genotype is associated with periodontitis in adolescents and has a high leukotoxicity. The aim of the study was to examine the prevalence of *Aa* and its JP2 genotype, the prevalence of the oral, commensal *Aggregatibacter aphrophilus* in a Maasai adolescent population, and the effect of herbal plants for inhibition of leukotoxicity. Methods: A total of 284 adolescents from Maasai Mara, Kenya, underwent an oral examination and microbial sampling. The presence of *Aa* and *A. aphrophilus* was analyzed by quantitative PCR and cultivation (the 58 samples collected at the last day of field study). The collected *Aa* strains were characterized and leukotoxin promoter typed. Additionally, herbal plants commonly used for oral hygiene were assessed for the inhibition of leukotoxicity. Results and Conclusions: The prevalence of *Aa* in stimulated whole saliva was high (71.8%), with the JP2 genotype detected in one individual, and *A. aphrophilus* in 99% of the sampled individuals. The commonly used herbal plant, *Warburgia ugandensis*, inactivated *Aa* leukotoxicity. The *Aa* virulence might be reduced through use of *W. ugandensis* and the high levels of *A. aphrophilus*.

## 1. Introduction

*Aggregatibacter actinomycetemcomitans* is a Gram-negative, facultative anaerobic bacterium frequently found in the oral cavity [1]. Although the bacterium has been shown to be strongly associated with periodontitis in adolescents, it can also exist in the oral cavity without causing disease [1,2]. Moreover, occasionally, this species is also associated with cases of extraoral diseases, such as endocarditis and rheumatoid arthritis [3,4]. The leukotoxin produced by the bacterium has recently been shown to be associated with rheumatoid arthritis [4]. Earlier studies on the association between periodontitis and *A. actinomycetemcomitans* have shown that the JP2 genotype, which has the ability to produce high amounts of leukotoxin, has a very strong association with periodontitis [5,6]. A typical characteristic of the JP2 genotype is a 530-basepair [bp] deletion in the promoter of the *ltxCABD* operon, encoding the leukotoxin [7]. The leukotoxin expressed by *A. actinomycetemcomitans* has been shown to not only kill leukocytes, but also to activate neutrophil degranulation, to protect the bacterium from phagocytic killing, and to initiate pro-inflammatory death of human macrophages [8,9,10]. These properties of the leukotoxin are associated with the cellular and molecular mechanisms involved in the pathogenicity of periodontitis [11]. Whereas periodontitis associated with *A. actinomycetemcomitans*, especially in adolescents, has been studied in northern and western parts of Africa, not much is known about the presence of *A. actinomycetemcomitans* in eastern parts of Africa [12]. Some studies performed in Sudan have indicated a rather high degree of rapidly progressing forms of periodontitis [13,14] associated with *A. actinomycetemcomitans* in the Sudanese population. However, no JP2 genotype of *A. actinomycetemcomitans* was identified [14]. Another Sudanese study discovered the JP2 genotype in one patient, who exhibited periodontitis with rapid progress [15], but the overall results from these examinations supported the notion that the presence of the JP2 genotype in East Africa is rather low [12]. Interestingly, an *A. actinomycetemcomitans* isolate sampled from a patient of Ethiopian origin living in Sweden was found to have an atypical 640-bp deletion in the leukotoxin gene promoter [16]. Whether this genotype might have originated from East Africa is not known. To the best of our knowledge, only one investigation on the topic was published about 30 years ago, which assessed the prevalence of periodontitis in adolescents in Kenya and reported a low prevalence of the disease compared to the studies carried out in Sudan [13,14,15,17]. The lack of microbial analyses in that study prompted us to investigate the presence of periodontal pathogens and other factors that could influence the susceptibility to periodontitis in Kenyan adolescents. The studied population in the present work consisted of adolescents living in Maasai Mara North Conservancy, Kenya, and has been described earlier [18].

Previous studies, which examined the presence of *A. actinomycetemcomitans* in relation to the occurrence of periodontal attachment loss, have included analyses of subgingival plaque samples [2,19]. However, a recent study examined the salivary presence of the JP2 genotype of *A. actinomycetemcomitans* in Moroccan adolescents positive for the JP2 genotype that has previously been detected in the subgingival plaque [19,20]. The JP2 genotype of *A. actinomycetemcomitans* could also be detected in saliva from all the 22 participants in the study, with the highest levels of the JP2 genotype in individuals with attachment loss (≥3 mm). This indicates that saliva is a suitable source to use for the examination of the presence of *A. actinomycetemcomitans* and its JP2 genotype on an individual basis.

Plants and natural products are widely used globally as substitutes for toothbrushes due to the cost, availability, customs, and religious reasons. Many of the plants used for oral hygiene purposes in Africa as well as other parts of the world have been shown to have antimicrobial properties against oral bacteria, including periodontal pathogens [20,21]. Among plants, *Psidium guajava* has been efficient in neutralizing the activity of the *A. actinomycetemcomitans* leukotoxin [22]. The most common herbal plants used by the population living in Maasai Mara, Kenya, have recently been reviewed [23]. In Maasai Mara, the use of plants for oral hygiene is common and includes a variety of different species [24], but the effects of these plants on the oral microbiota in this population has, however, not been studied intensely. In some urban and rural areas of the world, there is limited or no access to dental care and related products. It is therefore of particular importance to identify appropriate tools easily available for use in these populations.

*Aggregatibacter aphrophilus* is closely related to *A. actinomycetemcomitans*, when comparing the gene content. While *A. aphrophilus* as well as *A. actinomycetemcomitans* can be found in cases of endocarditis, it is not associated with periodontitis [25]. Considering that there is a large genetic similarity between these two bacterial species, it cannot be excluded that they compete with each other to proliferate in the same ecological niche in the oral cavity [3].

The primary aim of the present study was to examine the salivary presence, levels, and genotypic/phenotypic characteristics of *A. actinomycetemcomitans* and *A. aphrophilus* in a population (*n* = 284) of students at primary and secondary schools in the Maasai Mara area of Kenya. The secondary aim was to assess local factors that may potentially reduce the virulence of *A. actinomycetemcomitans*, such as anti-leukotoxic effects from herbal plants used for oral hygiene.

## 2. Materials and Methods

### 2.1. Study Population

**Figure 1:**
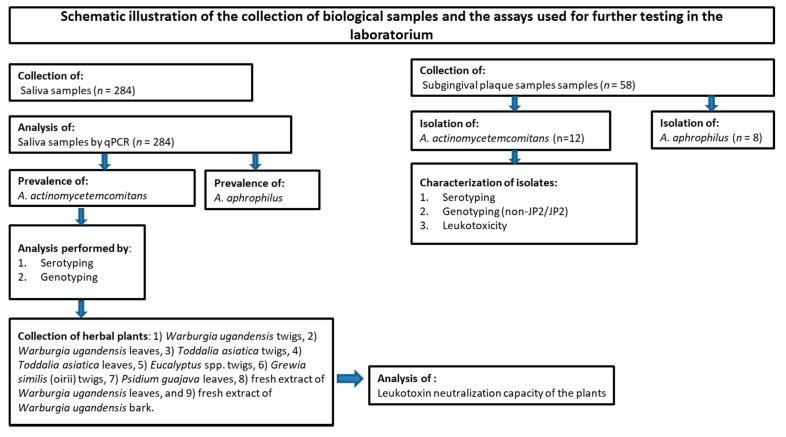
Flowchart describing the outline of the present work, assessing an adolescent population in Mara North Conservancy, Kenya (*n* = 284).

The study population consisted of 284 school children, aged 14 to 18 years (mean age: 15.0; SD 1.1; range 14–18 years), from five schools located in the Mara North Conservancy, Narok County, Kenya. The study population has previously been described in detail [18]. A schematic overview of the present study is shown in Figure 1.

### 2.2. Description of Field Conditions for Biological Sampling

Oral examinations were executed under field conditions in an ordinary classroom at the respective schools of the children in the Mara North Conservancy during five full working days. Subjects were made to lie on top of a table, facing a natural light source. A supplementary light source, a headlamp, was used to augment the natural light during the examination of the oral cavity. Using clean disposable mouth mirrors and tweezers, an oral examination was carried out to detect the status of the dentition. The methods and data from the clinical examination, as well as the field conditions, have been described previously; however, none of the clinical data are reported on in the present study [12,18].

### 2.3. Sampling of Stimulated Whole Saliva

The participants (*n* = 284) were asked to chew on a piece of paraffin wax for one minute, and the stimulated whole saliva was thereafter collected in a disposable plastic cup. One ml of the saliva was mixed with an equal volume of Saliva DNA Preservation Buffer (2X) (Norgen Biotek Corporation, Thorold, ON, Canada) in a 2 mL sterile tube and stored at room temperature until the DNA was isolated. This procedure was done prior to the oral examination made for other purposes [18].

### 2.4. Sampling of Subgingival Plaque for Microbial Cultivation

From a subgroup of the study population (*n* = 58; participants included on the last day of the field study at schools), dental plaque was collected with sterile paper points and inserted subgingivally in four periodontal pockets (mesial periodontal pockets on the 4 first permanent molars) for 10–20 s. The paper points from each patient were pooled into a tube with 2 mL of VMGAIII [26] transport medium supplemented with Nystatin (2 mg/L).

### 2.5. Isolation of DNA from Stimulated Whole Saliva

Five hundred µL saliva in buffer was mixed with 500 µL 10 mM Tris buffer with 1 mM EDTA (pH = 8.0) in a 1.5 mL Eppendorf tube and placed in an extraction instrument (Diasorin, Dublin, Ireland). DNA was extracted from 550 µL of the sample mixture with the Viral DNA extraction kit (Diasorin, Dublin, Ireland) with an elution volume of 100 µL in accordance with the protocol of the manufacturer. The samples were stored at +4 °C until the analyses were performed.

### 2.6. Quantification of A. actinomycetemcomitans and A. aphrophilus by qPCR

The loads of *A. actinomycetemcomitans* in the samples were quantified by qPCR using a Corbett Research Rotor Gene™ 6000 Real-Time PCR Thermocycler (Qiagen, Valencia, CA, USA). Specific primers and PCR cycling conditions used were as previously described [27]. The qPCR mixtures (10 µL) for the quantification of *A. actinomycetemcomitans* contained 5 µL Kapa Sybr Green (KK 4601) (Kapa Biosystems, Boston, MA, USA), 4 µL template, and 1 µL of the specific primer mix (0.5 µmol/L each). Each run included three negative samples (H_2_O) and standard mixtures with a given concentration equivalent to 10^1^; 10^2^, 10^3^, 10^4^, 10^5^, 10^6^, 10^7^, and 10^8^ *A. actinomycetemcomitans* cells/mL were prepared as described for the samples. The detection limit for the bacterium was set to 100/mL. *A. aphrophilus* loads in the samples were determined using the same general set up, however, using 3 µL template and 2 µL of the specific primer mix (0.5 µmol/L each) in the qPCR reactions, and with each run including one negative sample (H_2_O). The oligonucleotide primers used were a forward (5′-CCTACACCAGCGTTTATTTC-3′) and a reverse (5′-CTGAGGTTTACGCCAGTC-3′) primer, targeting an *A. aphrophilus*-specific gene sequence, encoding a putative hemolysin co-regulated protein (ACS98147; CP001607; [28]).

### 2.7. Sero- and Genotyping of A. actinomycetemcomitans in Stimulated Whole Saliva Samples

Samples containing ≥10^4^ *A. actinomycetemcomitans* cells/mL were analyzed with primers specific for serotype b of *A. actinomycetemcomitans* according to a previously described method [29]. Serotype b-containing samples were further analyzed for the presence of the JP2 genotype of *A. actinomycetemcomitans* by using leukotoxin promoter-specific oligonucleotide primers, as described previously [30]. PCR amplicons corresponding to the size (bp) of the JP2 genotype were isolated from agarose gels and their DNA sequences determined as described previously [16].

### 2.8. Detection of A. actinomycetemcomitans and A. aphrophilus in Plaque Samples by Cultivation

For the detection of *A. actinomycetemcomitans* in the dental plaque samples (*n* = 58) collected for cultivation from periodontal pockets of permanent first molars, aliquots (100 µL) were spread on a species-specific agar medium described by Slots, with the exception that the serum was omitted [31]. For the inhibition of growth of contaminating bacteria, i.e., enterobacteria, the samples were also spread on a medium modified as described by Höglund Åberg et al. [32]. The plates were incubated at 37 °C in aerobic atmosphere containing 5% CO_2_ for 3–5 days. Isolates from all *A. actinomycetemcomitans*-positive subjects (*n* = 12) were collected. The *A. actinomycetemcomitans* JP2 genotype reference strain, named HK1651, was included for comparison [33]. As reference, using the same approach, a smaller collection of *A. aphrophilus* strains (*n* = 8) were isolated from selected subjects. These isolates are referred to as 4-Aap-K, 12-Aap-K, 13-Aap-K, 21-Aap-K, 29-Aap-K, 30-Aap-K, 32-Aap-K, and 53-Aap-K, and have been found to exhibit resistance to human serum at a level similar to *A. actinomycetemcomitans* strains [34].

### 2.9. Characterization of A. actinomycetemcomitans Isolates

For serotyping, suspensions of the isolates were taken through a shaking block-heater at 95 °C for eight minutes and centrifuged. The supernatants obtained were used as a template and a PCR-based method described by Höglund Åberg et al. [32] was used. For the determination of the leukotoxicity, the isolates were cultured on peptone yeast extract agar at 37 °C in aerobic atmosphere containing 5% CO_2_ for 48 h, and the bacteria were harvested into 300 mM NaCl in phosphate buffered saline (PBS). The density was adjusted to OD 600 nm = 10 (≈10^10^ cells/mL), and the mixture was agitated at 4 °C for 60 min. The cells were pelleted by centrifugation (10,000× *g* for 10 min at 4 °C). The cell-free supernatant (5%) was added to cultures of phorbol 12-myristate 13-acetate (PMA)-differentiated THP-1 cells for 120 min, and cell lysis was determined by quantification of the leakage of LDH from damaged cells [35]. The release of LDH was expressed as % of the maximal release (100%) caused by incubation with 0.1% Triton x-100.

### 2.10. Collection and Extraction of Herbal Plants

Material from six different plants was collected in the Maasai Mara region of Kenya in collaboration with local experts. Plant species collected were (1) *W. ugandensis* twigs, (2) *W. ugandensis* leaves, (3) *Toddalia asiatica* twigs, (4) *T. asiatica* leaves, (5) *Eucalyptus* spp. twigs, (6) *Grewia similis* (oirii) twigs, (7) *Psidium guajava* leaves, (8) fresh extracts of *W. ugandensis* leaves, and (9) fresh extract of *W. ugandensis* bark. The *P. guajava* were not used in Maasai Mara but was included as a positive control based on previous findings [22]. The plant material was disintegrated and mixed with 70% EtOH (250 mg/mL) and agitated at room temperature for 24 h. The insoluble material was removed by centrifugation (5000× *g* for 20 min), and the supernatants were analyzed in leukotoxin neutralization assays.

### 2.11. Determination of the Leukotoxin Neutralization Capacity of Herbal Plants

The supernatants (1%) were added to cultures of PMA-differentiated THP-1 cells in the presence of leukotoxin (200 ng/mL) for 120 min, and cell lysis was determined by quantification of the leakage of lactate dehydrogenase (LDH) [36]. Purified leukotoxin was obtained by gel filtration of surface extracts from NaCl-treated cultures of JP2 genotype *A. actinomycetemcomitans* cells as described previously [9]. The release of LDH was expressed as percentage (%) of the maximal release (100%) caused by incubation with 0.1% Triton x-100. The ability of each extract to inhibit leukotoxicity was registered as a decrease in leukotoxin-induced cell lysis.

### 2.12. Statistical Analyses

A one-tailed paired *t*-test with Excel (Microsoft, Redmond, WA, USA) was used to determine any significant differences between samples. The confidence interval was set at 95% (*p*-value 0.05).

## 3. Results

### 3.1. Presence of A. actinomycetemcomitans and A. aphrophilus in Saliva Samples Determined by qPCR

**Figure 2:**
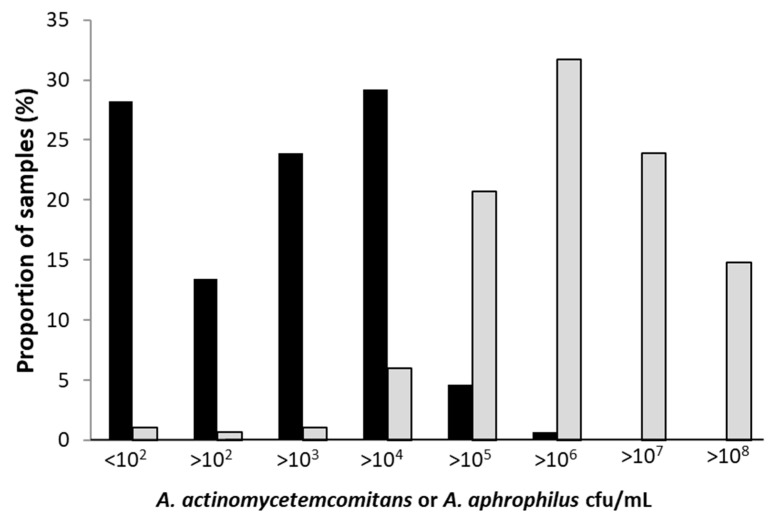
Proportions of saliva samples with indicated concentrations (cfu/mL) of *A. actinomycetemcomitans* (black bars) and *A. aphrophilus* (gray bars), respectively, as determined by qPCR. Samples from a total of 284 individuals of the Maasai Mara adolescent population were analyzed.

**Figure 3:**
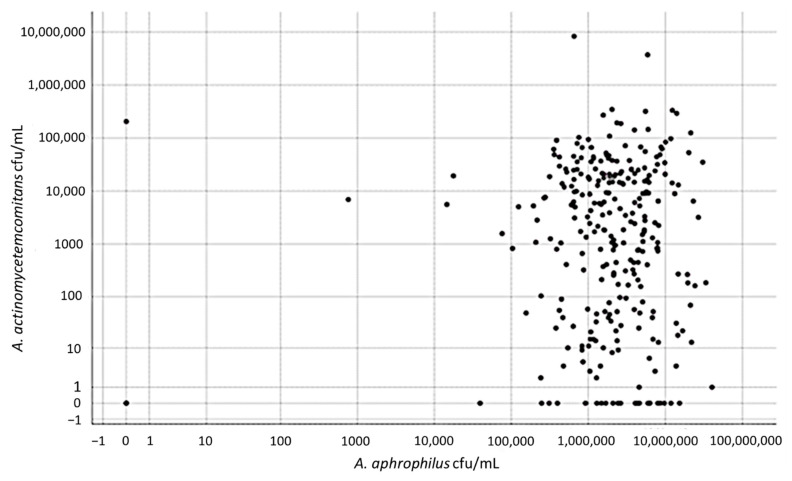
A comparison of the levels (cfu/mL) in saliva of *A. actinomycetemcomitans* and *A. aphrophilus*, respectively, as determined by qPCR. Samples from a total of 284 individuals of the Maasai Mara adolescent population were analyzed.

The prevalence of *A. actinomycetemcomitans* in the collected 284 stimulated whole saliva samples was 71.8%, with 204 out of the 284 analyzed saliva samples containing ≥100 cells of this species per mL. The distribution of the bacterium in specific concentration groups is shown in Figure 2. The prevalence of *A. aphrophilus* was higher, and in the 284 analyzed saliva samples, 99% (*n* = 282) contained ≥ 100 cells of this organism per mL. The distribution of the bacterium in specific concentration groups illustrates the generally higher loads of *A. aphrophilus* in the saliva samples relative to the levels of *A. actinomycetemcomitans* (Figure 2). This difference was emphasized by plotting the concentrations of *A. aphrophilus* against the concentrations of *A. actinomycetemcomitans* in the respective samples, revealing clearly higher levels of the former organism (Figure 3). In a few outliers (*n* = 2), however, we observed that there were instead high levels of *A. actinomycetemcomitans* relative to *A. aphrophilus*.

### 3.2. Sero- and Genotyping of A. actinomycetemcomitans in Stimulated Whole Saliva Samples

**Figure 4:**
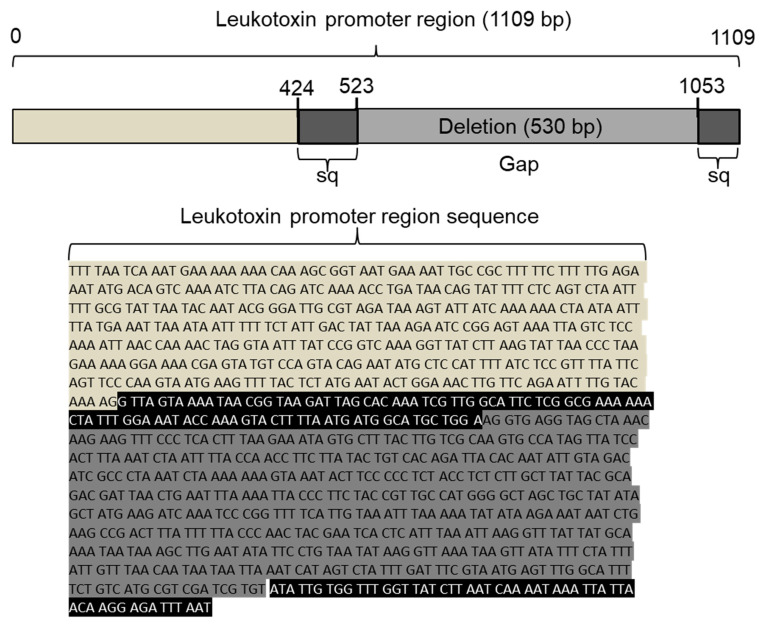
Schematic illustration of the leukotoxin promoter region. Extracted DNA from saliva of individuals positive for *A. actinomycetemcomitans* strains of serotype b was amplified by PCR using leukotoxin promoter-specific primers. The PCR products were visualized on an agarose gel, and one DNA band corresponding in size to that of the JP2 genotype was extracted from the gel and sent for DNA sequencing. The obtained sequence (highlighted in black) revealed the absence of the specific 530 base pair (bp) fragment (highlighted in dark gray), which is a specific characteristic of the JP2 genotype of *A. actinomycetemcomitans*.

When the 98 samples (34.5%) containing ≥10^4^ *A. actinomycetemcomitans* cells/mL were studied with regard to serotype b strains, 15 samples (16.5%) were found to contain this serotype. Saliva samples with <10^4^ *A. actinomycetemcomitans* cells/mL could not be serotyped with a reliable result. The subsequent leukotoxin promoter typing showed that 1 of the 15 samples had the 530-bp deletion, which is a characteristic of the JP2 genotype of *A. actinomycetemcomitans* (Figure 4).

### 3.3. Presence of A. actinomycetemcomitans in Subgingival Plaque Samples Determined by Cultivation

Samples from 58 individuals were cultured on agar plates, and *A. actinomycetemcomitans* could be isolated from 12 (22.1%) of 53 cultivable samples. It was not possible to examine five plates of the total number of samples due to overgrowth of other microbes. total of 11 (92%) of the 12 individuals, where *A. actinomycetemcomitans* could be detected by cultivation of the plaque sample, had >3000 bacterial cells/mL of this species in the saliva sample. Serotyping showed the presence of three different serotypes, five serotype a (41.7%), four c (33.3%), and three f (25%) (Table 1). Thus, no *A. actinomycetemcomitans* strains of the JP2 genotype of serotype b were identified by cultivation methods. *A. aphrophilus* was isolated from the plaque samples from eight individuals, who all carried a level of this species >10^2^/mL in their corresponding saliva sample.

### 3.4. Characterization of Cultivated A. actinomycetemcomitans Isolates

**Figure 5:**
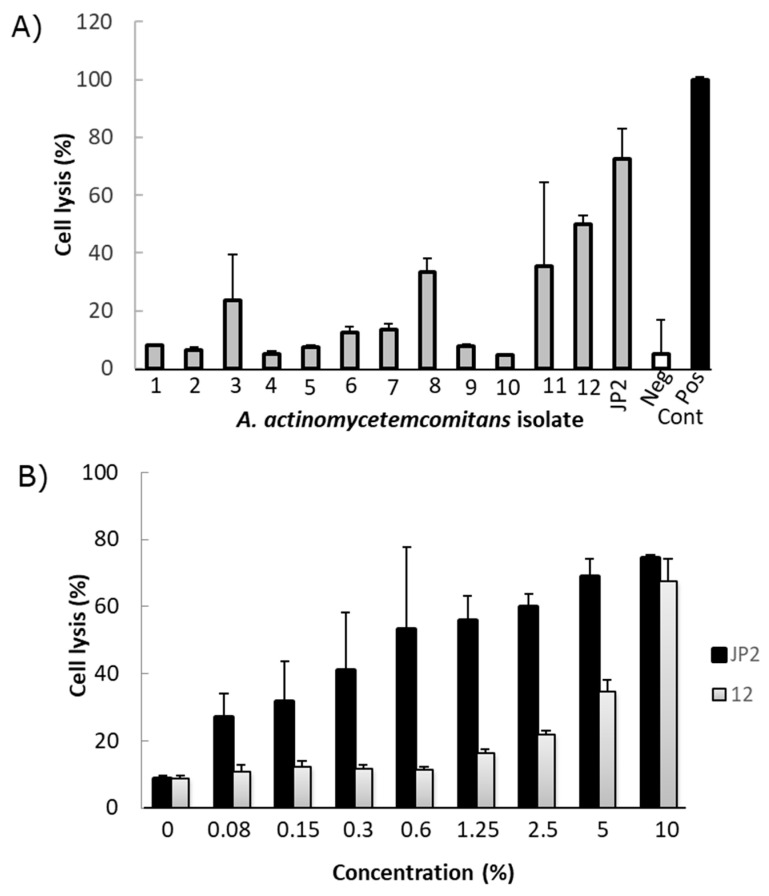
Leukotoxic activity of extracts from 12 different isolates of *A. actinomycetemcomitans* and a JP2 genotype strain of *A. actinomycetemcomitans* as comparison. (**A**) Effect on cell lysis in cultures of THP-1 cells exposed for 2 h. Gray bar is test bacteria, white bar is negative control and black bar is cell lysate. (**B**) Dose-dependent leukotoxicity in cultures of THP-1 cells exposed for 2 h in extract from isolate 12 (grey bars) and the JP2 genotype (black bars) of *A. actinomycetemcomitans*. Mean ± SD of triplicate analyses.

Leukotoxicity analyses of the *A. actinomycetemcomitans* isolates showed the presence of both low and intermediate leukotoxic phenotypes (Figure 5A). The isolate with the highest leukotoxicity (isolate 12; serotype a), as determined by the use of the LDH release assay (10), was compared with the JP2 genotype strain, HK1651, in a dose–response test. This indicated a substantial difference between the JP2 genotype and the selected isolate, which exhibited a lower leukotoxicity than the JP2 genotype (Figure 5B). However, none of the isolated *A. actinomycetemcomitans* strains were from serotype b or with the 530-bp leukotoxin promoter deletion.

### 3.5. Effects of Herbal Plants on Leukotoxic Activity

**Figure 6:**
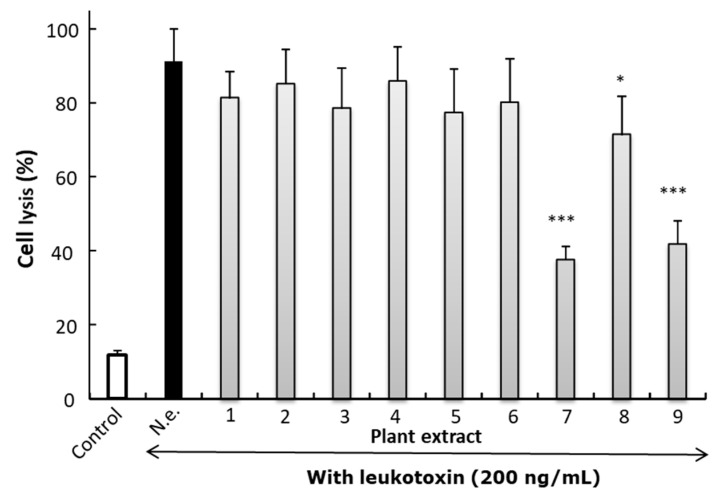
Neutralization of leukotoxicity in the presence of different plant extracts (1%) on cell lysis in cultures of THP-1 cell exposed for leukotoxin (200 ng/mL) in 2 h. N.e. No extract, (1) *W. ugandensis* twigs, (2) *W. ugandensis* leaves, (3) *T. asiatica* twigs, (4) *T. asiatica* leaves, (5) *Eucalyptus* spp. twigs, (6) *G. similis* (oirii) twigs, (7) Guava (*P. guajava)* leaves, (8) fresh extract of *W. ugandensis* leaves, and (9) fresh extract of *W. ugandensis* bark. White bar without leukotoxin, black bar with leukotoxin and gray bars leukotoxin + plant extract. Mean ± SD of four experiments analyzed in triplicates. Student’s *t*-test was used to examine significant difference of leukotoxicity without plant extract (*p <* 0.05 *. *p <* 0.001 ***).

**Figure 7:**
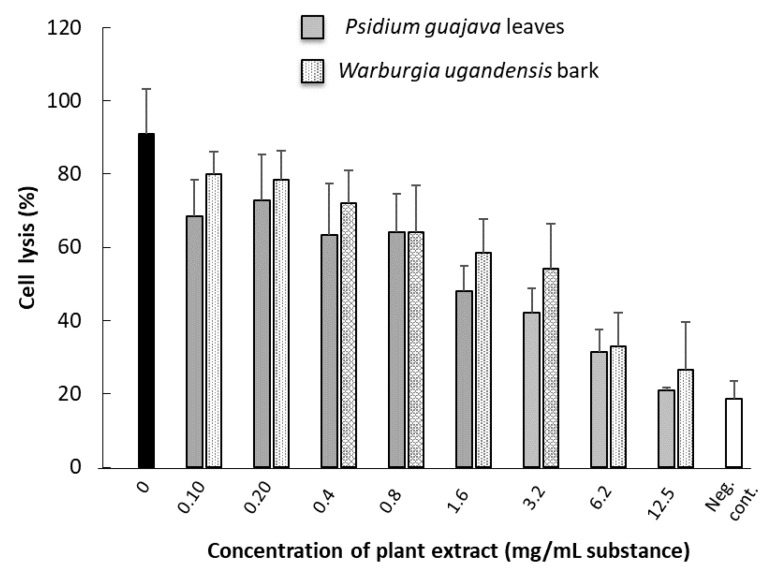
Dose-dependent effect on leukotoxic activity in the presence of extracts from *P. guajava* leaves or fresh extracts from *W. ugandensis* bark. Black bar with leukotoxin without plant extract. Mean ± SD of 3–6 observations from two separate experiments. Cultures of THP-1 cells were exposed for leukotoxin (200 ng/mL) for 2 h.

**Figure 8:**
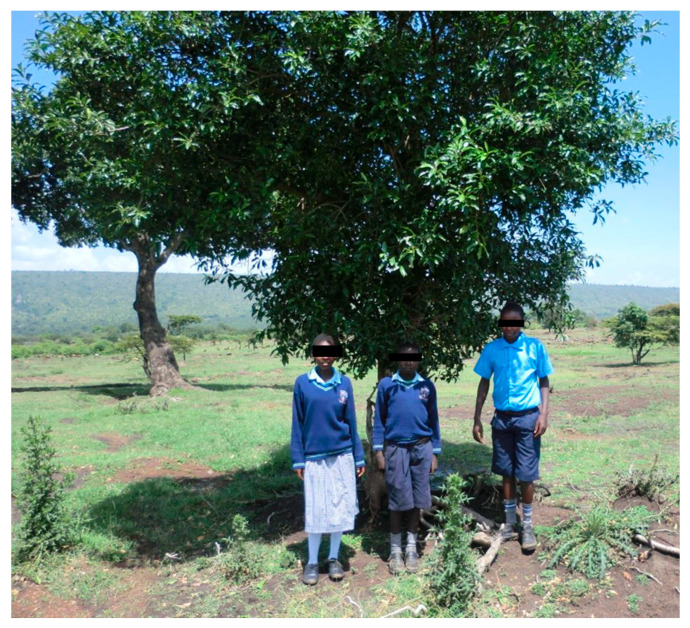
Pupils from the Mara Rianda boarding school standing in front of the *W. ugandensis* tree that they visit every morning to take a small branch to use as a chewing stick.

The ability of plant extract to inhibit leukotoxic activity was significant only for the extracts based on *P. guajava* leaves or fresh leaves or bark from *W. ugandensis* (Figure 6). It was shown by dose–response analyses that the neutralizing capacity of *W. ugandensis* bark extract was at a similar level as that of *P. guajava* leaves (Figure 7). Based on oral communications with examined pupils and their teachers, all individuals at the research site used *W. ugandensis* daily for oral cleaning. The pupils at the different schools could easily identify the tree from where they picked their chewing stick material (Figure 8).

## 4. Discussion

In the studied Kenyan Maasai adolescent population (*n* = 284), the salivary presence of *A. actinomycetemcomitans* was relatively high, with 71.8% of the population identified as carriers of the bacterium. This determined presence is in parity with other studies from far eastern countries [36,37,38,39], and with high carriage rates compared to those of 20–25% in western countries [40,41,42]. In a study from Sudan, the presence of *A. actinomycetemomitans* in subgingival plaque was high (70.6%) in cases diagnosed with rapidly progressing periodontitis, while this bacterium was only sporadically detected (5.9%) in the healthy controls [43].

Both the presence (99%) and loads of *A. aphrophilus* in the present work was found to be high among the examined Maasai adolescents, indicating that this bacterium is a conserved member of the normal oral flora in this population. In comparison with the salivary loads of *A. actinomycetemcomitans*, the concentrations of *A. aphrophilus* were substantially higher. Although not much is known regarding the presence of *A. aphrophilus* in populations worldwide, as this species has not been frequently assessed, our observations are consistent with some studies on populations in western Europe [44]. As *A. aphrophilus* presumably has low virulence potential related to the development of periodontitis, it could be hypothesized that it is beneficial to have high levels of this bacterium, which might allow lower numbers of *A. actinomycetemcomitans* to colonize in the same niche.

Interestingly, the detection of the JP2 genotype of *A. actinomycetemcomitans* in the present study population confirmed the previously reported observation [12]. By sequencing the leukotoxin promoter operon, we found the characteristic 530-bp deletion originally described by Brogan et al. [7]. A few *A. actinomycetemcomitans* isolates (*n* = 12) could be cultivated from samples of subgingival plaque; however, none of them were of the JP2 genotype of the bacterium. Further characterization of these isolates showed substantial leukotoxic activity in a few of them, but at levels lower than those exhibited by the JP2 genotype of *A. actinomycetemcomitans* strains.

The dissemination pattern of the JP2 genotype of *A. actinomycetemcomitans* has been examined in previous studies by analyses of subgingival plaque samples [5,32,45]. This strategy requires a stable transport medium, sterile paper points, and instruments to handle the paper points relatively fast, if cultivation is desired [26]. Hence, in the present study, this procedure was performed only with the 58 adolescents sampled on the last day of the field study in order to avoid a too long transportation time of the bacterial samples from the relatively remote research site in Maasai Mara, Kenya, to the laboratory at Umeå University, Umeå, Sweden.

In comparison, saliva, which is easily collected, showed reproducible results over time, and when frozen, it can retain the DNA stable for long periods of time [46,47]. Saliva has also been proven to display comparable results with subgingival plaque samples [48]. This makes saliva more suitable for large population studies, which would also facilitate the possibility of determining the dissemination pattern of the JP2 genotype of *A. actinomycetemcomitans*. Since *A. actinomycetemcomitans* can be found at different sites in the oral cavity [49], we could also expect to find *A. actinomycetemcomitans* more frequently, in comparison to when subgingival plaque samples are analyzed.

The plant used for oral hygiene measures by the population of Maasai school pupils, *W. ugandensis*, was found in the area surrounding the schools. As we showed in our experimental setup, the plant extracts had a neutralizing effect on the leukotoxin produced by *A. actinomycetemcomitans*. This could potentially have an influence on the presence of periodontitis and the virulence capacity of *A. actinomycetemcomitans* in this area, where this plant is frequently used. We are aware of the limitation of the present study, as the periodontal status was not examined for most of the individuals included. Therefore, no cause–effect relationship could be determined. The potential anti-bacterial effects of the plant towards other oral bacteria are not known but would be relevant to assess, as such effects might contribute to preventing the shift in the oral microbial ecology towards a more periodonto-pathogenic composition.

## 5. Conclusions

In conclusion, we, by the assessment of the adolescent population in Maasai Mara, found the JP2 genotype of *A. actinomycetemcomitans*, serotype b for the first time in Kenya. While the reported prevalence of periodontitis in Kenya is low, our finding of a relatively high presence of *A. actinomycetemcomitans* in the examined population could indicate the influence of virulence-modifying factors that may counteract this species. This might include factors such as substances in oral hygiene tools from herbal plants and/or in the commensal oral flora.

## Figures and Tables

**Table 1:** Plaque samples for cultivation were collected from 58 of the students examined on the last day of the planned field study. Five samples were discarded due to overgrowth of bacteria and yeast. In 53 of the 58 plaque samples collected, *A. actinomycetemcomitans* could be detected and isolated in 12 (22.6%) of them. These 12 isolates were serotyped and examined for their leukotoxic activity.

Number	Aa Isolate	Serotype
1	Aa-3-K	f
2	Aa-4-K	f
3	Aa-6-K	c
4	Aa-11-K	a
5	Aa-17-K	f
6	Aa-23-K	c
7	Aa-25-K	c
8	Aa-29-K	a
9	Aa-30-K	a
10	Aa-38-K	c
11	Aa-51-K	a
12	Aa-52-K	a

## Data Availability

Microbiological data are stored by AJ at the University of Umeå, Sweden.
